# Natural Ingredients Common in the Trás-os-Montes Region (Portugal) for Use in the Cosmetic Industry: A Review about Chemical Composition and Antigenotoxic Properties

**DOI:** 10.3390/molecules26175255

**Published:** 2021-08-30

**Authors:** Sara Gonçalves, Isabel Gaivão

**Affiliations:** Department of Genetics and Biotechnology and CECAV, University of Trás-os-Montes and Alto Douro, 5000-801 Vila Real, Portugal; sgoncalves@utad.pt

**Keywords:** almonds, antigenotoxic, cosmetics, elderberry, genotoxicity, grapes, natural ingredients, olives

## Abstract

The natural cosmetics market has grown since consumers became aware of the concept of natural-based ingredients. A significant number of cosmetics have an ecological impact on the environment and carry noxious and chemically potent substances. Thus, the use of natural and organic cosmetics becomes increasingly important since it is clear that topical treatment with cosmeceuticals can help improve skin rejuvenation. A substantial investigation into the benefits that fruits and plants can bring to health is required. Studies have shown that antigenotoxic properties are linked to anti-aging properties. Several studies have shown potential antigenotoxicity in natural ingredients such as Almonds (*Prunus dulcis*), Elderberry (*Sambucus nigra*), Olives (*Olea europaea*), and Grapes (*Vitis vinifera*). This review presents an overview of research conducted on these natural ingredients, the most common in the Northeast of Portugal. This region of Portugal possesses the most organic farmers, and ingredients are easily obtained. The Northeast of Portugal also has climatic, topographic, and pedological differences that contribute to agricultural diversity.

## 1. Introduction

Aging is a physiological process that affects all structures of the organism, with the particularity of each organ and tissue having its own rhythm of aging [1]. It is characterized by the progressive inability to maintain vital functions, it is harmful, and it is considered to be the final stage of human development, ending with death. Oxidative stress is believed to be a significant factor for speeding up the process of aging [2]. Although several theories have been proposed to justify aging, it is a multifactorial process. It involves the cumulative effects of extrinsic influences and an intrinsic molecular program of cellular aging. According to stochastic theories of aging, free radical atoms are unstable due to the loss of electrons and are continuously produced by body metabolism. Damage occurs when they react violently with other molecules in the cell. They can affect cell maintenance and repair if the damage occurs on DNA. So, we grow old because of cumulative damage to our body cells from external and internal sources [1]. The aging of the skin is primarily associated with the intrinsic genome. However, diet, lifestyle, drug and alcohol history, and environmental exposures are other factors that influence skin aging by affecting DNA [3]. On the other hand, deterministic theories maintain that aging is a direct consequence of a genetic program; the genome and molecular structure are a type of molecular clock [1].

There are many ways in which we are exposed to toxic substances: through the air we breathe, the food we eat, the water we drink, the clothes we wear, cosmetics, radiation exposure, which also has harmful effects. Toxic substance exposure is much more problematic today than it would have been in the past. The environmental repercussions include DNA damage, and this genome instability leads to diseases such as cancer, degenerative diseases, infertility, diseases associated with aging [4], among many other issues. A healthy lifestyle can reduce these issues, including consuming substances that protect the genome by several mechanisms reducing DNA damage. Genotoxicological studies are fundamental for knowing the hazards to genome and health, and antigenotoxicological studies are the answer to minimize genome instability.

The ability of different agents to produce damage to genetic material is called genotoxicity. The agents capable of causing genetic toxicity are classified into three categories, according to their origin: physical, which includes ionizing and electromagnetic radiation, temperature, and ultraviolet light; chemical, consisting of heavy metals, pesticides, aromatic hydrocarbons, alkylating agents, acridine, acrylamide, aliphatic epoxides, organic solvents, asbestos particles, food additives and xenobiotics; and biological, such as parasites, bacteria, plants, viruses and fungi [4,5].

The term cosmetics, as defined by the current European regulation on cosmetics, refers to a product applied to the body to beautify, cleanse, or improve the appearance and enhance attractive features [6]. Included in the definition of cosmetics are soaps, shampoos, toothpaste, cleansing and moisturizing creams for regular care, color cosmetics, hair colorants, and styling agents, fragrance products, and ultraviolet light (UV light) screening preparations [7]. Although conventional, natural and organic cosmetics have the same definition, they differ in their specificities. The formulation of conventional cosmetics does not need to contain certified natural and organic ingredients [8]. Natural cosmetics is a product that must have at least one ingredient “derived from” some natural substance, extracted directly from a plant or mineral rather than being synthesized. Natural cosmetics can contain percentages of organic ingredients. However, a natural product is not necessarily an organic product [9]. An organic cosmetic must contain at least 95% of certified organic ingredients in its composition. These raw materials are obtained through certified crops and extraction. They must be biodegradable and preserve the most natural chemical characterization. The remaining 5% of the formulation may be composed of water, natural raw materials from agriculture, or non-certified allowed extractive for organic formulation [7,8,9].

Modern-day cosmetics increasingly include noxious and chemically potent substances in modern days and have an ecological impact on the environment [10]. As awareness of this grows, people tend to buy organic and natural products more frequently. Analysts maintain an optimistic long-term outlook on the global natural and organic personal care products market. It is a market that has grown and amounts to €288.9 million in 2021. The market is expected to grow annually by 9.24% (2021–2025) [11,12,13]. A total of 80% of French women buy or have already bought natural and organic beauty products [14]. About 50% of French consumers decided to buy organic cosmetics after realizing the ecological impact of non-organic products [15,16]. The organic cosmetic market grew by 5.9% in Germany, reaching 1.26 billion EUR in 2018 [17].

Thus, the use of natural and organic cosmetics becomes increasingly essential. A study performed by the Danish Council THINK Chemicals found 65 chemicals of concern in 39 products. The major results of this study are listed in Table 1 [18].

Consumers exposed themself to these chemicals, perhaps daily. Since the genotoxic agents are present in many cosmetics, a substantial investigation into the benefits that plants and fruits can bring to health is required since it is clear that topical treatment with cosmeceuticals can help improve skin rejuvenation [25]. Identifying products with antigenotoxic effects is among the most promising research areas in recent years since they might protect against DNA damage and its consequences. Studies have shown that antigenotoxic properties are linked to anti-aging properties [4,26], and these properties are essential to revert genotoxic effects. Therefore, this review presents an overview of research conducted on natural ingredients common in the Northeast of Portugal with an antigenotoxic effect.

## 2. Characterization of Trás-os-Montes Region

Trás-os-Montes is limited to the west by the province of Minho, to the south by the Douro, to the east by the Douro River, and to the north by Spain. The climate is sub-Atlantic/continental and Mediterranean, represented by “Terra Fria de Planalto”, “Terra Fria de Montanha”, “Terra Fria de Alta Montanha”, “Terra de Transição” and “Terra Quente”. The climate is influenced in the west by the humidity from the Atlantic, in the east by the cold and dryness from the continent, and in the south by the heat [27]. The ecological zoning of Trás-os-Montes highlights the domains: Atlantic (50%), Iberic (26%) and Mediterranean (24%) and four agrotypes, “Granito e Xisto,” “Meia Encosta Nordestina,” “Terra Fria Transmontana” and “Terra Quente Transmontana” [28]. Physiography is dominated by mountain and sub-mountain hypsometry, from 450–700 m (natural limit of vine culture) to 1500 m [29]. The dominant soils are of granitic origin and the like, associated with schist [30]. In the Valleys of Vila Pouca de Aguiar, Chaves, Vila Real, and Boticas (<700 m), there is a greater variety of crops, such as vines, olive trees, fruit trees, wheat, potatoes, rye, maize, and permanent pastures [27]. This review will focus especially on Elderberry, Almonds, Olives, and Grapes.

Elderberry is commonly found in the north, especially in the Varosa Valley, which because of the surrounding mountains, produces a microclimate favorable for the development of this species [31,32]. The almond tree is one the most widely planted tree crops in the Trás-os-Montes area, occupying an area of 19,206 hectares [33]. The most common varieties are “Parada”, “Casanova”, “Verdeal” and “Pegarinhos” [34]. Trás-os-Montes is the second Portuguese olive growing region, currently representing between 12 and 15% of the national production of olive oil [35]. The more important varieties are “Cobrançosa”, “Madural” and “Verdeal” [36]. Portugal has a wine-growing area of 1/4 to 1/5 of the surface of the significant wine-growing countries in Europe. Of the 343 grape varieties listed, about 230 varieties are considered indigenous to Portugal or the Iberian Peninsula, reflecting the vast and unique Portuguese viticultural genetics. Trás-os-Montes area has 40 indigenous varieties in cultivation [37]. As such, natural ingredients are easily obtained in this area [38]. It is also a region with the most organic farmers, and the climatic, topographic, and pedological differences predispose this region for agricultural diversity [16].

## 3. Natural Ingredients from Northeast of Portugal

### 3.1. Elderberry (Sambucus nigra *L*.)

Overview: The Elderberry is a species of the *Caprifoliaceae* family. It grows in most parts of Europe, North Africa, West Asia, and the USA [39]. The flower blossoms generally in May or June [40]. The fruit matures over six to eight weeks from July to September [41].

Flowers and berries have a large spectrum of applications such as cosmetics, for skin and eye lotions; in the kitchen, for liquors, wine, jelly, and chutneys; in the food industry, as a natural dye; and in medicine, for cold, flu and phlegm [42]. Various studies suggest that Elderberry helps to reduce enhanced production of inflammatory mediators [43], has beneficial effects on blood pressure [44], diabetes, and obesity [45,46], has antiviral, antibacterial, and antifungal activity [47], protects against UV radiation [48] and has laxative and diuretic activity [49]. Tree leaves and barks present diuretic and healing properties. They should only be used for external use since they cause poisoning due to cyanogenic glycosides, the most common being sambunigrin and prunasin. They also contains m-hydroxysubstituted glycosides, such as zierin and holocalin. These compounds are toxic and life-threatening because they can be hydrolyzed, resulting in the release of cyanide [46,50].

#### 3.1.1. Chemical Composition

Chemical composition varies depending on the cultivar, development stage, ripening, and season. As for carbohydrates, elderberry berries contain 7.86–11.50% of total sugar and 2.8–8.55% of reducing sugar. The main sugars are glucose (33.33–50.23 g/kg FW) and fructose (33.99–52.25 g/kg FW). Sucrose is also found in small amounts (0.47–1.68 g/kg FW) [51]. Other carbohydrates were found such as dietary fibre, in particular, pectin (0.1593%), pectin acid (0.2299%), protopectin (0.0409%), Ca-pectate (1.53%), and cellulose (1.65%) [52]. As for proteins, they are present in berries (2.7–2.9%), flowers (2.5%), and leaves (3.3%). Thus protein includes sixteen amino acids, nine of which are essential (9% in flowers and 11.5% in leaves) [51]. Glutamic acid (0.311 g/100 mL), aspartic acid (0.303 g/100 mL) and alanine (0.238 g/100 mL) were reported as the dominant amino acids, whereas cysteine (0.008 g/100 mL), methionine (0.025 g/100 mL) and histidine (0.062 g/100 mL) where less abundant [52]. Fats are accumulated mostly in elderberry seeds (22.4%, with 75.15% of polyunsaturated fatty acids and 14.21% of monounsaturated fatty acids) and seed flour (15.9%, with 21.54% of polyunsaturated fatty acids and 4.21% of monounsaturated fatty acids) [51]. Organic acids represent 1.0–1.3% of the berry content. Four organic acids were detected in elderberry fruit. The most dominant was citric acid (3.08–4.81 g/kg FW), followed by malic acid (0.97–1.31 g/kg FW) and smaller concentrations of shikimic (0.14–0.93 g/kg FW) and fumaric acid (0.10–0.29 g/kg FW) [53]. Minerals are located both in berries and flowers and represent 0.90–1.55% of the fruit mass. They include K (391.33 mg/100 g), P (54.00 mg/100 g), Ca (28.06 mg/100 g), Na (2.17 mg/100 g), Mg (25.99 mg/100 g), Fe (1.86 mg/100 g), Zn (0.36 mg/100 g), Mn (0.27 mg/100 g) and Cu (0.14 mg/100 g) [52]. Elderberry fruit and flowers also include essential oils (0.01%), consisting of approximately 53 compounds in berries and 58 in flowers [51]. Vitamin C and cellulose are also found in elderberry fruit in concentrations of 34.10 mg/100 g and 1.65 mg/100 g, respectively [52].

Elderberry fruit has components with high biological activity, primarily polyphenols and anthocyanins. The major polyphenols in elderberry fruit are chlorogenic acid, neochlorogenic acid, crypto-chlorogenic acid, quercetin, quercetin-3-rutinoside (rutin), quercetin-3-glucoside (isoquercitrin), kaempferol-3-rutinoside, kaempferol-3-glu-coside (astragalin), isorhamnetin-3-rutinoside and isorhamnetin-3-glucoside [51]. The primary flavonol in this plant is rutin, while isoquercitrin and astragalin occur in elderberries in smaller amounts [54]. Elderberry also have quercetins in their composition: quercetin (2.70–4.50 mg CGE/100 g FW), quercetin 3-rutinoside (35.88–50.04 mg CGE/100 g FW) and quercetin 3-glucoside (6.38–26.52 mg CGE/100 g FW) [53]. As for anthocyanins, five were found in elderberry fruit: cyanidin 3-sambubioside-5-glucoside (19.52–53.49 mg CGE/100 g), cyanidin 3,5-diglucoside (7.41–23.29 mg CGE/100 g)), cyanidin 3-sambubioside (270.8–630.8 mg CGE/100 g), cyanidin 3-glucoside (221.4–586.4 mg CGE/100 g) and cyanidin 3-rutinoside (1.49–9.63 mg CGE/100 g) [53]. Elderberry fruit contains small amounts of tannins. These are procyanidins, like epicatechin (88.4% of total tannins) and catechin (11.6% of total tannins) and their thiol derivatives [51].

Elderberry fruit also has high antioxidant activity. According to Duymuş et al. [55], elderberry extract shows antiradical activity (towards DPPH), having an IC_50_ value (concentration needed to scavenge 50% of free radicals) of 123 µg/mL. Espín et al. [56] demonstrated lower radical scavenger capacity towards DPPH than other studied sources of anthocyanins and natural and synthetic antioxidants. Obied et al. [57] also investigated the antioxidant potential of Elderberry, with results indicating that elderberry fruit can protect colon cells against harmful effects of oxidative stress. Elderberry berries have a vibrant chemical composition. According to Imenšek et al. [58], the consumption of 100 g of berries could cover 13% of the recommended daily intake of calcium for women and men. The cellulose content is vital for lowering the risk of developing type II diabetes and heart diseases. Elderberry has antioxidant and anticancer activity thanks to the high content of vitamin C and anthocyanins. It has a protective influence in many chronic degenerative diseases due to the high protein level and seven essential amino acids [52]. Therefore, using fruits, flowers and leaves may constitute a potential protective agent against growth and unfavorable effects of oxidative stress in the human body [54,59].

Elderberry flowers contain much higher amounts of phenolic compounds than the fruits and leaves [54]. The main phenolic compounds found are chlorogenic acid, neo-chlorogenic acid, cryptochlorogenic acid, 3- and 5-feruloyl quinic acid, and dicaffeoylquinic acid. As for flavonols, glycosides of quercetin, kaempferol, and isorhamnetin are present in elderflowers [60]. Another group present in elderflowers is flavanols, including catechin, epicatechin and procyanidin trimer, and flavanones [61]. They also possess higher antioxidant activity than berries and leaves. A study performed by Kołodziej and Drożdżal showed that the time to reduce DPPH concentration by 50% was 23–75 s for flowers and fruit 91–133 s. A study performed by Imenšek et al. [58] analyzed the macro and micronutrients in various parts of the Elderberry plant. Elderberry leaves have the highest calcium content (1.38 ± 0.04%) and significantly higher magnesium content (0.73 ± 0.05%) than other plant parts. Phosphorus was also found but in significantly lower proportions (0.30 ± 0.02%). As for micronutrients, iron (115 ± 4 mg/kg DW), manganese (67.0 ± 4.6 mg/kg DW), zinc (39.7 ± 2.0 mg/kg DW), and strontium (35.0 ± 2.2 mg/kg DW) were detected.

#### 3.1.2. Potential Application in the Cosmetic Industry

Elderberry possesses ingredients favorable for cosmetic formulation, such as anthocyanins, which that can reduce oxidative stress by scavenging free radicals, making them a potential anti-aging agent [62]. Elderberry flowers are potential sources of active substances, which in contrast to the antioxidants commonly added to cosmetics, are not subjected to degradation under the influence of ultraviolet radiation and exhibit a high biological activity [48,63]. Limonene and linalool, monoterpenic compounds, are often employed in perfumes, creams, and soaps [64]. Cellulose enhances hydration’s physical and structural properties and the skins’ oil holding capacity [65]. 

Relevant studies of Elderberry: Only two studies have been developed since 2012, showing that Elderberry has no mutagenic effect and a high in vitro activity (Table 2). 

### 3.2. Olives (Olea europaea *L*.)

Overview: Olives are the fruit of the Olive tree, a species of the *Oleaceae* family. The olive tree is one of the most ancient cultivated fruit trees, and the use of Olives has been ascertained in the late Stone Age at the Kfar Samirin site in Israel [68]. In the Graeco-Roman civilization, olive oil and wine were closely associated due to the similarities in their transformation process and economic importance. They were used not only in daily life but also in trade, religious rites, and art. Since prehistoric times, the olive tree has been of significant cultural importance in that region and still has symbolic and religious significance today [69]. Olive trees are usually distributed in the coastal areas of the eastern Mediterranean basin, the contiguous coastal areas of south-eastern Europe, northern Iran at the south end of the Caspian Sea, western Asia, and northern Africa [70]. The best olive oil should be acidic [71], from the first cold pressure, preferably from of organic farming [36]. 

#### 3.2.1. Chemical Composition

The main constituents of the olive flesh are water (60–75%) and lipids (10–25%) [72]. Olives have a lower sugar content (2–5%). The main sugars in the flesh are glucose, followed by fructose, galactose, and mannitol [72,73]. The protein content of the fresh pulp is relatively low (1–3%), and its amino acid composition does not differ significantly [72]. The fruit also contains flavonoids, mainly luteolin, apigenin, quercetin-3-rutinoside (rutin), and anthocyanins. Some of these compounds can be found in olive oil and may contribute to its antioxidant properties [74]. Significant components of crude fiber (1–4%) are cellulose, lignin, and hemicellulose. Olives are considered a rich source of phenolic compounds (1–3%) with a wide array of biological activities with significant nutritional, physiological, and pharmaceutical effects on human health. [75]. The primary polyphenols found in raw olives are oleuropein, verbascoside, ligstroside, salidroside, rutin, luteolin-7-glucoside, cyanidin-3-glucoside, and cyanidin-3-rutinoside [72]. Ash varies from 0.611%, with the significant elements being K, Ca, P, Na, Mg, and S [76]. Organic acids are found in concentrations between 0.5% and 1%, which are malic and citric acids, the primary acids in raw olives [72].

Olive leaves are a source of bioactive compounds, particularly polyphenols and consist of simple phenols, flavonoids, and secoiridoids [77,78]. The concentration levels of main phenolic compounds in olive leaves are as follows: for secoiridoids, oleuropein aglycone (14.8 × 10^3^ mg/kg), oleuropein glucoside (6600 mg/kg), demethyloleuropein (2300 mg/kg), oleuropein (6.97 × 10^3^–441 × 10^3^ mg/kg), ligstroside (12,400 mg/kg), oleuroside (2010–7000 mg/kg), methoxyoleuropein (870–2190 mg/kg), oleoside (10,800 mg/kg), and secologanoside (7300 mg/kg); for flavonoids, and in the flavones group there is present luteolin (10.1–5600 mg/kg), luteolin glucoside (507–10,500 mg/kg), luteolin diglucoside (0.0–121.4 mg/kg), luteolin rutinoside (67–2700 mg/kg), apigenin (1–480 mg/kg), apigenin glucoside (12–680 mg/kg), apigenin diglucoside (90–480 mg/kg), apigenin rutinoside (7.3–1130 mg/kg), diosmetin (1–37 mg/kg), and chrysoeriol-7-Oglucoside (580–840 mg/kg); as for flavonols, rutin (13.8–3500 mg/kg), quercetin rutinoside (654–1210 mg/kg), and quercetrin (1–129 mg/kg); as for flavan-3-ols, catechin (0.8–64.2 mg/kg); for simple phenols, tyrosol (90–660 mg/kg), tyrosol glucoside (860–1280 mg/kg), hydroxytyrosol (30.8–11,400 mg/kg), and hydroxytyrsol glucoside (340–790 mg/kg); for phenoli aldehyde, vanillin (1.3–8.2 mg/kg); for phenoli acids, vanillic acid (12.8–110.1 mg/kg), caffeic acid (1.60 mg/kg), gallic acid (7.4–55.8 mg/kg), cinnamic acid (5.4–44.5 mg/kg), hydroxycinnamic acid (5040–32.69 × 10^3^ mg/kg), syringic acid (174–447 mg/kg), ferulic acid (7–91.4 mg/kg), verbascoside (29 × 10^3^ mg/kg), isoverbascoside (17,200 mg/kg), p-hydroxybenzoic acid (0.6–23.8 mg/kg), cholorogenic acid (3.4–3.8 mg/kg), protocatechuic acid (2.3–61.0 mg/kg), and hydroxyphenylacetic acid (14.7–45.7 mg/kg). Other compound are also present such as elenolic acid (99.6–662.927 mg/kg), elenolic acid glucoside (5600 mg/kg), and elenolic acid diglucoside (270–1370 mg/kg) [78,79]. Olive leaves have antibacterial properties and can be used to treat wounds [80]. They are also diuretic and, as such, used to relieve joint pain, and gout [81]. They are also febrifuges and hypotensive [82,83], as well as having have anti-diabetic properties, helping to lower blood sugar [84]. Olives have widely been exploited as a functional food [62,85] with various biophenols [57].

According to the “Autoridade de Segurança Alimentar e Económica” (Food and Economic Safety Authority) in Portugal, the standard grades of olive oil currently available on the market are extra virgin olive oil, virgin olive oil, and “azeite lampante” [86]. The standard grades are based on the free acidity or degree of processing of the oil. The free acidity for extra virgin olive oil is ≤0.8%, virgin olive oil ≤ 2.0%, and “azeite lampante” ≥ 2.0%. Olive oil has minerals, such as calcium (1 mg/100 g), iron (0.56 mg/100 g), potassium (1 mg/100 g), and sodium (2 mg/100 g); vitamins, namely vitamin E (14.35 mg/100 g) and vitamin K (60.20 µg/100 g); and lipids, saturated fatty acids (15.40 g/100 g), monounsaturated fatty acids (69.20 g/100 g), and polyunsaturated fatty acids (9.07 g/100 g) [73,87]. Olive oil has diverse fatty acids, namely myristic, palmitic, palmitoleic, heptadecanoic, heptadecenoic, stearic, oleic, linoleic, linolenic, arachidic, eicosenoic, behenic, and lignoceric acids [79]. One of the major hydrocarbons present in olive oil is squalene. Squalene appears to be critical for reducing free radical oxidative damage to the skin and has been used as a moisturizing or emollient agent in cosmetic preparation [88].

Olive oil is rich in molecules with antioxidant and anti-inflammatory functions, such as ω-3 polyunsaturated fatty acids, ω-9 monounsaturated fatty acids, and phenolic compounds [89]. Olive oil is recommended in the diet of pregnant women as it favors the healthy development of the brain and nervous system of the baby before and after birth [90,91]. It also allows better bone mineralization [92]. Olive oil prevents the accumulation of fats in the liver, lowers blood pressure, prevents atherosclerosis, and prevents thrombosis [93,94,95,96]. Olive oil can benefit another group of dementia-related pathologies called tauopathies [97]. It could also prevent diseases related to oxidative damage, such as coronary heart disease, stroke, and several types of cancer [98]. Olive oil could also have an anti-aging effect. Olive oil is widely used in soaps and massage oils [99]. It is an excellent vehicle for macerating aromatic plants and flowers for therapeutic and culinary uses.

#### 3.2.2. Potential Application in the Cosmetic Industry

Virgin olive oil provides a safe and stable emulsion delivery system [100]. The antioxidant activity of olives makes them a candidate for moderating the effects of the aging process on the skin by limiting biochemical consequences of oxidation [101] due to its high squalene content and β-sitosterol, and richness in oleic acid (a skin softener). As such, virgin olive oil is ideal for directly protecting the skin [102]. Oleuropein is used in cosmetics due to its antioxidant, antiviral, antimicrobial, anti-inflammatory, skin protecting, and anti-aging properties [65]. Fatty acids increase hydration, softness and elasticity and act as a protective barrier [65,103].

Relevant studies of Olives: Several studies have been developed since 2005, showing that Olives have an antigenotoxic effect, antioxidant potential, protect cells from induced DNA damage, and minimize cytotoxicity (Table 3).

### 3.3. Almonds (Prunus dulcis)

Overview: The Almond tree is a species of the *Rosaceae* family. It is the oldest nut crop of southwest Asia, and from that region it has diffused to other regions and continents [106]. Hippocrates was the first to discuss the use of almonds for cold and other phlegmatic disorders [107]. Due to the successive Greek, Roman, and Arab invasions, almond cultivation spread in a narrow horizontal band westward through the Mediterranean to Spain [106]. Almonds can be eaten as dried fruit and are also used for pastry and liquors. The almond shell is used for biofuel [108].

#### 3.3.1. Chemical Composition

Almonds have a composition of 4.70 g/100 g of water, 21.22 g/100 g of protein, 3.89 g/100 g of total sugar, 12.30 g/100 g of dietary fibers, and 49.42 g/100 g of total fat; this represents 46–64% of fat, composed of monounsaturated fat, such as oleic acid (58.1–71.3%), polyunsaturated fat, linoleic acid (15.7–29.9%) and saturated fatty acids, such as palmitic (5.9–7.5%), stearic (0.1–2.4%), arachidic (0.07–0.10%), and myristic (0.02–0.05%) acids [109]. As for proteins, their levels can vary from 10% to 35%. Amino acids have a residual content in the ripe kernel (<200 mg/100 g), such as lysine and arginine [110]. Carbohydrates are present in almond kernels (2–12%). 90% of the total sugars are sucrose and raffinose, whereas the remaining 10% are dietary fibers, mainly cellulose, hemicelluloses, and lignin [111]. Almond kernel is considered a good source of minerals. The major elements are K, P, Ca, and Mg, while the minor elements are Fe, Na, Cu, Zn, and Mn [112]. Tocopherol is also present in the almond kernel. A study showed that the content of α-tocopherol could vary from 139 to 355 mg/kg [113], representing 97.3% of total content, whereas γ-tocopherol represents 2.8% [114]. As for phytosterols, an average value of total phytosterol amount in almond kernel ranges from 1100 to 2800 mg/kg, β-sitosterol being the principal component [115]. Phenolic compounds are also found in the almond kernel, catechin’s main phenolic compound, followed by chlorogenic acid and naringenin [110]. Almond skin is also rich in phenolic compounds, such as (+)-catechin, (−)-epicatechin, naringenin-7-*O*-glucoside, kaempferol-3-*O*-rutinoside, isorhamnetin-3-*O*-rutinoside, isorhamnetin-3-*O*-glucoside, and naringenin. The flavonoids present are isorhamnetin, isorhamnetin-3-*O*-glucoside, kaempferol, quercetin-3-galactoside, catechin, kaempferol-3-*O*-glucoside, kaempferol-3-*O*-galactoside, and quercetin [110,116]. Also, some alcohols (1-butanol, 1-hetanol, 1-hexanol, 1-nonanol, 1-octanol, 1-pentanol, 1,2-propanediol, 2-ethyl-1-hexano, 2-heptanone, 2-methyl-1-propanol, 2-phenylethanol, 3-methyl-1-butanol, 3-methyl-2-buten-1-ol, 3-methyl-3-buten-1-ol, and benzyl alcohol), acids (acetic and hexanoic acids), pyrazine (2-methylpyrazine), terpene (α-pinene and limonene), lactone (butyrolactone), alkane (toluene), and aldehydes (benzaldehyde, heptanal, hexanal, nonanal, octanal, and pentanal) have been described being present in raw almonds [117,118]. Squalene is also present in almonds (95.0 mg/kg) [114]. The consumption of Almonds has been linked to reduced risk of chronic diseases such as coronary heart disease and type 2 diabetes and weight maintenance and control [109]. Almonds are a source of energy and macronutrients and are high in fibers. Almonds are also rich in chemically related phytosterols, which help maintain healthy blood cholesterol levels [109].

#### 3.3.2. Potential Application in the Cosmetic Industry

Sweet almond oil is used in the cosmetic industry, especially in dry skin creams, anti-wrinkle and anti-aging products. It enhances the glow and fairness of the skin. It is used in over 280 cosmetic formulations in concentrations up to 50% [119]. When mixed with white wine and honey, it can be applied to urticaria and wound healing [120]. Almond oil is one of the most popular oils used in aromatherapy and massage therapy since it is suitable for any skin type. Since it contains large amounts of vitamins E and K, it helps skin regeneration and elasticity [121].

Relevant studies of Almonds: Table 4 shows the results of antigenotoxic studies of Almonds. 

### 3.4. Grapes (Vitis vinifera)

Overview: Grapes are the fruit of the Winegrape, a species of the *Vitaceae* family. It is one of the most consumed fruits globally, mostly in juice and wine, but some are destined for fresh consumption or dried into raisins [127,128]. The archaeological record suggests that cultivation of the domesticated grape, *Vitis vinifera* subsp. *vinifera* began 6000–8000 years ago in the Near East from its wild progenitor, *Vitis vinifera* subsp. *sylvestris* [128]. After pruning in January, clusters are formed in the spring, and it is during the summer that the Grapes gain color, aroma, and taste. Between September and October, when the Grapes are already ripe, that is when their weight, color, and acidity present the ideal conditions for wine production, the harvests take place [129].

#### 3.4.1. Chemical Composition

Grape skin is rich in flavonoids (myricetin-glucuronide, myricetin-glucoside, quercetin-glucuronide, quercetin-glucoside, and kaempferol-glucoside) and anthocyanins (such as delphinidin-monoglucoside, cyanidin-monoglucoside, petunidin-monoglucoside, among others), with a total amount of 4863 mg/kg and 1670 mg/kg, respectively [130]. Phenolic acids are also present in the seeds, skin, and pulp. The ones found are gallic acid, protocatechuic acid, catechin, epicatechin, procyanidin, epigallocatechin, resveratrol, chlorogenic acid, coumaric acid, caffeic acid, and rutin. Organic acids and sugars are also present in seeds, skin, and pulp, the major ones being tartaric, malic, and citric acids, glucose, and fructose [131].

Grapeseeds contain 8–20% of oil and are a by-product of the winemaking process, and their oil content is traditionally extracted using either mechanical techniques or an organic solvent. Cold-pressing is a method of oil extraction involving no heat or chemical treatment and may retain more health-beneficial components [132,133,134]. The total amount of polyphenols extracted from grapeseed oil by the cold-pressing method is about 2.9 mg/kg. They are a rich source of catechins and procyanidins [135]. Grapeseed oil contains many phenolic compounds, including flavonoids, carotenoids, phenolic acids, tannins, and stilbenes. However, the polyphenols content in grape seed oil is low (0.013–0.019% of total phenolic compounds) [136]. They also contain palmitic, 11-eicosenoic, oleic, linoleic, α-linolenic, and arachidic acids [137]. Flavan-3-ols are also present in the seed, namely catechin (67.71 ± 11.37 mg/100 g) and epicatechin (57.93 ± 22.12 mg/100 g) [130]. *Trans*-resveratrol is also present in grapes (0.62 ± 0.24 mg/kg). Benzene compounds can also be found in grapes, the most abundant being benzyl alcohol (540–1140 µg/kg of grape) and homovanillic alcohol (380–960 µg/kg of grape). Grapeseed oil has a great vitamin E content, ranging from 1 to 53 mg per 100 g of oil [138]. Vitamin E contributes to has a high antioxidant activity and neuroprotective and antitumoral properties [139,140]. For this reason, grape seed oils have been suggested to delay the aging process and prevent some chronic diseases. Grapeseed oil has antioxidant, anti-inflammatory, antimicrobial, and antitumoral properties [132,136,139,141]. It has also been used as edible oil. 

In winemaking, a large amount of grape pomace is produced that is rich in polyphenolics and highly beneficial for human health, as phenols are helpful for skin ultraviolet (UV) protection. Incomplete extraction of compounds such as polyphenols during winemaking leads to about 70% of the initial active substances remaining in grape pomace waste, 20–30% in peels, and 60–70% in seeds [142,143,144]. Some groups of polyphenols present in *V. vinifera L.* grape pomace are flavanols (flavan-3-ols), flavonols, anthocyanins, condensed tannins, and proanthocyanidins [145].

#### 3.4.2. Potential Application in the Cosmetic Industry

Grapes can be used in cosmetics as follows: bud extract, flower extract, fruit extract, fruit powder, fruit water, juice, juice extract, leaf extract, leaf oil, leaf/seed skin extract, leaf water, leaf water, leaf wax, root extract, seed, seed extract, seed powder, shoot extract, skin extract, skin powder, vine extract, and vine sap. The seed extract is used in 463 cosmetic formulations, fruit extract in 219 cosmetic formulations, and leaf extract is used in 78 cosmetic formulations [146]. Grape seeds contain fiber (40%), oil (16%), protein (11%), sugars, and minerals being a rich source of proanthocyanidins. Proanthocyanidins are potent antioxidants and have free radical scavenger activities [147]. The various components of grapes make them an excellent ingredient to be added to cosmetic formulations. Resveratrol’s proven ability to penetrate the skin barrier and anti-aging activity makes it an excellent complement for cosmetic formulation. It can also stimulate fibroblasts’ proliferation and increase the concentration of collagen III [148]. Phenolic acids and flavonoids, such as ferulic acid, caffeic acid, gallic acid, and proanthocyanidins, are efficient protectors by reducing oxidative stress and may be essential in cosmetic surgery formulation for post-sun skin care [149]. Grapes also provide phenolic components, like anthocyanins, gallic acid, catechin, epicatechin, conjugated flavonoids, oleic, linoleic, and linolenic acids, counteracting symptoms of epidermal aging and delaying the process of photoaging [150,151]. Currently, to optimize sun protection and photostability, sunscreens use natural antioxidant composition. Scientific evidence has shown the benefits of polyphenols’ topical and oral use from some plant species against UV radiation, including *Vitis vinifera* [120,121,146,152].Relevant studies of Grapes: Table 5 shows relevant studies developed on Grapes since 1999.

## 4. Discussion

Micronucleus, Comet Assay, and SMART (in *Drosophila melanogaster*) are widely used tests for genotoxicity of potential substances and detect DNA damage evidenced by strand breaks in various organisms and tissues. Excessive generation of free radicals may result in DNA damage. At least two major human problems, aging, and carcinogenesis, involve DNA damage. The possible protective effect of these natural ingredients against oxidative DNA damage should be investigated. We are exposed to noxious substances daily. Table 6 presents different assays used in genotoxicological studies, as well as the advantages and disadvantages.

These natural ingredients have been shown to possess a variety of biological activities and to hold therapeutic promise. Elderberry presents fewer studies in comparison with the other natural ingredients. The difficulty of accessing such natural ingredients in some countries and the fact that it is a wild tree can be some of the reasons for the lack of studies on this tree. On the other hand, grapes and olives have various in vivo and in vitro studies. Also, some of their studies have been performed in *D. melanogaster* and human lymphocytes. *D. melanogaster* is one of the preferred organisms for toxicological research. In recent decades, it has been used as a model to elucidate human diseases and also for toxicological studies [185,186,187]. The use of *D. melanogaster* in experimental studies met the standard of the European Centre for the Validation of Alternative Methods (ECVAM): reduction, refinement, and replacement (3Rs) of laboratory animal usage. *D. melanogaster* as a model raises few ethical concerns [188]. The short life cycle, the distinct developmental stages, the availability of various tools and reagents, known genome sequence, and the physiological similarity of *D. melanogaster* with humans (namely on dietary input, xenobiotic metabolizing system, antioxidant enzymes, and DNA repair systems) make them an excellent in vivo model organism to rapidly test toxicity in the whole organism and elucidate the molecular mechanisms underlying the toxicity [160]. In biomedical sciences, experimental evidence has implicated oxidative stress in the pathophysiology of several disease conditions [189]. However, the precise role of oxidative stress in the pathology of diseases is far from being known. Therefore, the study of oxidative stress in animal models is of particular importance at present. *D. melanogaster* has been used to assess oxidative stress and antioxidant markers [190,191]. They have been used in genotoxicity and antigenotoxicity studies. In vivo tests for detecting somatic or, germinative mutations are especially valuable.

Human lymphocytes are used as surrogate tissue, as they are easily obtained, are available in large numbers, do not require cell culture, are diploids, and are almost all in the same phase of the cell cycle. Although the Comet Assay is well accepted among the scientific community, there are issues regarding standardization among laboratories [192]. Therefore, new methods for DNA damage assessment would be beneficial to improve research on DNA damage repair and antigenotoxicity.

## 5. Conclusions

The present review synthesizes the most accurate evidence of the antigenotoxic capacity of some natural ingredients common in the Northeast of Portugal. Almonds, Grapes, Olives, and Elderberry proved to have an antigenotoxic effect. Natural occurring antigenotoxicity in natural ingredients could strongly counteract genome instability.

Even though these ingredients are already being used in cosmetics, the lack of antigenotoxicological studies makes it crucial to investigate further how to incorporate them in cosmetics to benefit human health. Studies have shown that plants, fruits, and vegetables with antigenotoxic properties show promising results for the cosmetic industry [193,194,195]. Additional investigation can be carried out, namely, evaluating the cosmetic properties of the natural ingredients towards promoting DNA integrity. Using Comet Assay and SMART, evaluating genoprotection, longevity, and prolificacy of the natural ingredients in *D. melanogaster* could reveal exciting results. 

## Figures and Tables

**Table 1:** Major chemicals found, their uses, and problematic properties.

Chemical	Uses	Problematic Properties	Comments	Reference
Iodopropynyl butylcarbamate	Preservative	Allergenic	The Cosmetic Ingredient Review concluded that IPBC was safe as a cosmetic ingredient at concentrations less than or equal to 0.1%, but IPBC should not be used in products intended to be aerosolized because of the negative effect on the lungs.	[19]
Butylparaben	A preservative used in personal care products	Endocrine disruptor	The available data for butylparaben show strong evidence that this compound has estrogenic effects in vivo in Uterotrophic assays performed in immature females. One in vivo study has shown adverse effects on sperm counts following perinatal exposure, while there are conflicting results on the influence of butylparaben on sperm count/quality following exposure of young male rats. Butylparaben causes vitellogenin induction in fish.	[20]
Resorcinol	Numerous uses, including rubber and resins, in cosmetics, pharmaceuticals and hair dye	Endocrine disruptor	Resorcinol has been shown to affect thyroid function as well as estrogen and glucose metabolism. According to human case reports, resorcinol exerts anti-thyroid functions. Resorcinol’s long-term administration to permeable (damaged) skin can cause myxoedema (reduced thyroid function). Cessation of exposure causes the myxoedema to disappear. In the human study investigating dermal uptake in healthy individuals, the dermal barrier avoids uptake of resorcinol.	[20]
Ethylhexyl Methoxycinnamate (OMC)	UV-filter	Endocrine disruptor	There is strong evidence that OMC can affect the endocrine system in vivo. Slight but significant increases in uterine weights have been seen in both intact immature and adult ovariectomized rats. In a 2-generation study, a significant decrease in sperm cell number was seen. In contrast, another reproductive study has shown developmental OMC exposure to cause several adverse reproductive effects in offspring, including reduced reproductive organ weights, reduced reproductive hormone levels, reduced sperm counts, and neurobehavioural effects. OMC can also interfere with the hypothalamo-pituitary-thyroid axis in vivo, as many studies have shown reduced levels of thyroxine in the blood. OMC affects the transcription of genes involved in hormonal pathways, including vitellogenin, in most fish studies.	[20]
Glyoxal	Preservative Antimicrobial	Mutagenic	As for the in vitro studies, Glyoxal is reported to be a mutagen in renaturation assays, unscheduled DNA synthesis (UDS) assays, the Ames assay, the Escherichia coli SOS chromotest, the Bacillus subtilis liquid rec-assay, the rat hepatocyte primary DNA repair test (single strand breaks found, but no DNA cross-linking), sister chromatid exchange assays, Chinese hamster ovary (CHO) and Chinese hamster V79 chromosome aberration assays, the CHO/HGPRT gene mutation assay (only with metabolic activation), the mouse lymphoma L5178y/TK+/− system, and in vivo in the rat, where UDS and increased alkaline elution of DNA were seen in glandular stomach tissue and single strand breaks in liver tissue DNA (not seen in kidney, spleen, pancreas, and lung). It was negative in the C3H/I0T1/2 cell transformation assay and the in vivo mouse micronucleus assay. For the in vivo studies, Glyoxal was mutagenic in most assays. Glyoxal inhibited the effect of DMN in a short-term oral study in rats.	[21]
Propylparaben	Preservative	Potential endocrine disruptor	Propylparaben is associated with estrogenic and antiandrogen activity, affecting sperm function and prenatal development, among others. The substance has been detected in biomonitoring studies and human urine and milk.	[22,23]
Zinc Pyrithione	Preservative Antimicrobial	CRM Category 1B	Zinc pyrithione, which has been classified as a category 1B carcinogen, is now prohibited for use in cosmetic products.	[24]

**Table 2:** Relevant studies of Elderberry.

Year	Main Objective	Type of Study	Assay Employed	Material	Conclusion	Reference
2012	Evaluate possible cytotoxic and genotoxic effects of anthocyanin-rich Elderberry concentrate	In situ	*Allium cepa* (mitotic index) Test	*Sambucus nigra* fruit extract powder	*S. nigra* fruit extract powder has a very high in vitro antioxidant activity and no mutagenic effects at low concentrations	[66]
2015	Detection and isolation of lectins from *S. nigra* flowers, and investigation of some of their biological properties with an emphasis on genetic effects	In vitro	*hprt* locus test (Chinese hamster cells) Disk diffusion method	Lectins from *S. nigra*	Shows a link of antimutagenic/mutagenic and protective effects of *S. nigra* flower lectins with the regulation of repair functions on the molecular level	[67]

**Table 3:** Relevant studies of Olives.

Year	Main Objective	Type of Study	Assay Employed	Materials	Conclusion	Reference
2018	Investigate the genotoxic and protective effects of fullerene C_60_ and Virgin olive oil in rats induced by cadmium chloride.	In vivo	Chromosomal aberrations in bone marrow cells from rats (*Rattus norvegicus)*	Virgin olive oil mixed with fullerene C_60_	Virgin olive oil is described as a potential antioxidant and protects cells from induced DNA damage by cadmium exposure	[104]
2019	Monitor the possible hazardous effects of hexavalent chromium on rats and the possible effects of extra-virgin olive oil against hexavalent chromium induced toxicity by studying the cellular alteration, DNA damage, immune function alterations, and histopathological changes of rat spleen	In vivo	Micronucleus assay and Estimation of serum 8-hydroxy-2-deoxyguanosine level in blood samples from rats (*Rattus norvegicus)*	Extra virgin olive oil obtained directly from olives, purchased from a local market	Extra-virgin olive oil succeeded in minimizing cytotoxicity and genotoxicity	[105]

**Table 4:** Relevant studies of Almonds.

Year	Main Objective	Type of Study	Assay Employed	Materials	Conclusion	Reference
2006	Study to test the efficacy of two different doses of Almonds in reducing oxidative damage and oxidative stress among smokers	In vivo	Alkaline Comet Assay with enzyme (endonuclease III) in human lymphocytes	Almond powder	Almond consumption is protective against oxidative stress and DNA damage among smokers	[122]
2011	Study potential genotoxicity of almond skins	In vivo	Micronucleus test and mammalian bone marrow chromosome aberration tests in Swiss Albino Mice (*Mus musculus)*	Almond skins that were turned into a brown powder	Almond skins are not genotoxic	[123]
		In vitro	Bacterial reverse mutation assay with *Salmonella typhimurium*			
2011	Study the effects on cell growth, cell cycle modulation as well as the genotoxic/anti-genotoxic potential of the fermentation supernatant of colon adenocarcinoma cells	In vitro	Alkaline Comet assay with enzyme (Fpg) in human colon adenocarcinoma cell line HT29	Purchased almonds digested in an in vitro simulation of the human gastrointestinal passage	Fermented nuts are considered to be not genotoxic in HT29 cells	[124]
2015	Investigate possible beneficial or harmful effects of Ultra high-pressure homogenization technology application on physical, nutritional, and bio-functional properties of almond milk	In vitro	Alkaline Comet assay with enzyme (endonuclease III) in human colon adenocarcinoma cell line Caco-2	Almond milk samples produced from almond powder	No significant differences between raw and Ultra high-pressure homogenization-treated almond milk indicating that Ultra high-pressure homogenization-treatment did not affect the genotoxic potential of almond milk	[125]
2017	Investigate both the chemopreventive effects of fermented raw Almonds	In vitro	Alkaline Comet assay (Basic assay) in human colon adenoma cell line LT97	In vitro digestion and fermentation of roasted almonds	The genotoxic potential of fermentation supernatants of roasted Almonds can be excluded even for the highest roasting temperature	[126]

**Table 5:** Relevant studies of Grapes.

Year	Main Objective	Type of Study	Assay Employed	Materials	Conclusion	Reference
2002	Study the influence of a great variety of food components, i.e., fruits, vegetables, spices, and beverages of plant origin, on the genotoxicity of aromatic amines	In vitro	Alkaline Comet Assay (basic assay) in rat (*Rattus norvegicus*) commercial cell line	Red and white wine and grape juice	Genotoxicity was strongly reduced in a dose-related manner by red Grapes	[153]
2004	Examine the antigenotoxic and protective effects of a procyanidin extract from grape seed	In vivo	Alkaline Comet Assay (basic assay) in the rat (*Rattus norvegicus*) commercial Fao cells	Procyanidin extract obtained from grape seeds	A complex mixture of wine polyphenols protected against some types of chemically induced oxidative DNA damage in the rat	[154]
2009	Study the genotoxicity of a commercial grape seeds proanthocyanidin extract alone and its antigenotoxic effects on Doxorubicin-induced somatic mutation and recombination in the wing spot test of *Drosophila melanogaster*	In vivo	Somatic mutation and recombination test (*D. melanogaster*)	Purchased grape seeds proanthocyanidins (Vittis^®^)	Grape seed proanthocyanidins were not genotoxic	[155]
2011	Assessment of the health-protecting properties of the skin, seeds, and pulp of *Vitis vinifera* fruit	In vitro	Somatic mutation and recombination test in *Drosophila melanogaster*	Skin, seeds, and pulp from grapes	Procyanidins are antigenotoxic, and flavonoids prevent the damage caused by mutagens in DNA	[156]
2014	Evaluate the antimutagenic and antigenotoxic potential of grape juice concentrate in rodent organs exposed to cadmium chloride intoxication	In vivo	Micronucleus test in the bone marrow and liver tissue from rats and Alkaline Comet Assay (basic assay) in peripheral blood and liver cells from rats (*Rattus norvegicus*)	Grape juice	Shows a significant reduction in DNA damage. Grape juice concentrate was able to protect liver cells against the oxidative stress by H_2_O_2_	[157]
2016	Evaluate the effect of unfermented grape juice on the levels of genomic damage in Chronic kidney disease patients under dialysis by analyzing markers such as genomic/oxidative DNA damage and chromosome damage	In vivo	Alkaline Comet Assay with enzyme (Fpg) and Micronucleus Test in human lymphocytes	Purchased unfermented grape juice	Significant decreases in the underlying levels of oxidative DNA damage was obtained	[158]
2017	Investigate the chemical composition of different parts of strawberry grape	In vivo	SOS chromotest (*Escherichia coli)*	Peel, seed, leaf, and stalk of strawberry grape	Seed and stalk extracts together with the leaves of the grape showed high antigenotoxic activity	[159]

**Table 6:** Assays used in genotoxicological studies.

Assay	Characteristics	Advantages	Disadvantages
Comet Assay	Sensitive and rapid method for DNA strand break detection in individual cells. Used in genotoxicological studies to determine oxidative DNA damage that has been implicated in several health conditions (in combination with certain bacterial enzymes), to show protective effects of different dietary factors in chemopreventive studies, to determine sequence or gene-specific damage and repair (in combination with fluorescence in situ hybridization) as well as of possible diagnostic use [160,161].	Identify DNA damage at the single-cell level Sensitivity for detecting low levels of DNA damage using a small number of cells per sample (<10,000) Eukaryote single cell population can be used both in vitro and in vivo Ease of application Low cost Short time needed to perform the assay [161,162]	Not a reliable biomarker for genotoxic effects in DNA damage The detected DNA damage does not correspond to fixed mutations [161,163,164,165]
Somatic Mutation and Recombination Test (SMART)	In vivo genotoxicity assays are performed on *Drosophila melanogaster.* It has been used extensively to analyze many chemicals with different action mechanisms, showing a vast ability to detect genotoxic effects. [160,166,167].	Easy to perform Inexpensive Shows high sensitivity, specificity, and accuracy Detects loss of heterozygosity by deletions, point mutations, mitotic recombination, and nondisjunction [166,168]	Time-consuming Delay in the development of the larvae [168,169]
Micronucleus Test	A small, chromatin-containing round-shaped body that is visible in the cytoplasm of cells. Can originate from acentric fragments, segregation of chromosomes, dicentric chromosome breakage, chromosome instability, or aggregation of double minutes. A rise in the frequency of micronucleated cells is a biomarker of genotoxic effect that can reflect exposure to agents with clastogenic (chromosome breaking; DNA as target) or aneugenic (aneuploidogenic; effect on chromosome number; mostly non-DNA target) modes of action. [160,170,171,172,173,174].	Reliable identification of cells that have completed only one nuclear division Easy to perform Less time required Inexpensive Allows the detection of chromosome and genome mutations and co-detection of apoptosis and necrosis, measuring the extent and progression of nuclear division in a dividing cell population Detects dicentric bridges, chromosome loss, nondisjunction, excision repair events, and Hypoxanthine guanine phosphoribosyl transferase variants Allows automatic scoring [175,176]	Low sensitivity Detects only acentric fragments Cell division is needed for effective use [176]
Chromosomal Aberration Test	It can be performed in primary peripheral blood lymphocytes or established cell lines, such as Chinese hamster ovary cells. Recognizes agents that cause structural chromosome aberrations (clastogenesis) produced by various mechanisms [177,178].	Allows identification of all chromosome mutation types and co-detection of mitotic indices [178]	Need for cell cultivation and highly skilled and experienced personnel Time-consuming Expensive Automatic scoring is not possible [176,179]
Bacterial Reverse Mutation Test	Uses amino acid-requiring strains of *Salmonella typhimurium* and *Escherichia coli* to detect point mutations, which involve substituting, adding, or deleting one or a few DNA bases pairs. Detects chemicals that induce mutations that revert mutations present in the tester strains and rehabilitate the functional capability of the bacteria to synthesize an essential amino acid. [180,181,182,183].	Allows replicates to be made and results to be obtained relatively quickly Allows inexpensive study of a large number of test materials inexpensively Enables identification of the molecular mechanism effect of test material Very versatile test [183,184]	May not be appropriate for the evaluation of certain classes of pharmaceutical, for example, highly bactericidal compounds such as antibiotics, and those which are thought (or known) to interfere specifically with the mammalian cell replication system Can produce false positives Possible interference from biological samples, e.g., plant extracts that contain amino acids (histidine) with the test system [180,184]

## Data Availability

Data available in a publicly accessible repository.

## References

[1] Pathath A.W. (2017). Theories of Aging. Int. J. Indian Psychol..

[2] Sram R.J., Binkova B., Rossner P. (2012). Vitamin C for DNA damage prevention. Mutat. Res. Fundam. Mol. Mech. Mutagen..

[3] Graf J. (2005). Anti-Aging Skin Care Ingredient Technologies. Cosmetic Dermatology.

[4] Izquierdo-Vega J., Morales-González J., SánchezGutiérrez M., Betanzos-Cabrera G., Sosa-Delgado S., Sumaya-Martínez M., Morales-González Á., Paniagua-Pérez R., Madrigal-Bujaidar E., Madrigal-Santillán E. (2017). Evidence of Some Natural Products with Antigenotoxic Effects. Part 1: Fruits and Polysaccharides. Nutrients.

[5] Nagarathna P.K.M., Wesley M., Reddy P., Reena K. (2013). Review on genotoxicity, its molecular mechanisms and prevention. Int. J. Pharm. Sci. Rev. Res..

[6] Singh S.K. (2010). Handbook on Cosmetics (Processes, Formulae with Testing Methods).

[7] EUR-Lex—31976L0768—EN (1997). Council Directive 76/768/EEC of 27 July 1976 on the Approximation of the Laws of the Member States Relating to Cosmetic Products. https://eur-lex.europa.eu/legal-content/EN/TXT/HTML/?uri=CELEX:31976L0768&from=EN.

[8] Romero V., Khury E., Aiello L.M., Leonardi G.R. (2018). Differences between organic and natural cosmetics: Clarifying literature for prescribers. Surg. Cosmet. Dermatol..

[9] Fonseca-Santos B., Corrêa M., Chorilli M. (2015). Sustainability, natural and organic cosmetics: Consumer, products, efficacy, toxicological and regulatory considerations. Braz. J. Pharm. Sci..

[10] Okereke J.N., Udebuani A.C., Ezeji E.U., Obasi K.O., Nnoli M.C. (2015). Possible Health Implications Associated with Cosmetics: A Review. SJPH Sci. J. Public Health.

[11] Natural Cosmetics: Sales Value France. Statista 2015. https://www.statista.com/statistics/671090/natural-organic-cosmetics-sales-value-france/.

[12] Global Market Value for Natural/Organic Cosmetics and Personal Care in 2018–2027. Statista n.d. https://www.statista.com/statistics/673641/global-market-value-for-natural-cosmetics/.

[13] Natural Cosmetics—Germany | Statista Market Forecast. Statista 2021. https://www.statista.com/outlook/cmo/beauty-personal-care/cosmetics/natural-cosmetics/germany.

[14] Natural and Organic Cosmetics: Share of Female Users France 2016. Statista 2016. https://www.statista.com/statistics/671247/natural-organic-cosmetics-share-women-france/.

[15] Organic Cosmetics: Decisive Moments for Consumption France 2016. Statista 2016. https://www.statista.com/statistics/671320/key-moments-consumer-organic-cosmetics-france/.

[16] Gonçalves S., Gaivão I. (2021). Evaluation of Cosmetic Properties of Natural Ingredients in the Trás-Os-Montes Area: A PhD Project. Sci. Posters.

[17] Germany: Natural and Organic Cosmetics Gained More Than a Million New Customers in 2018. Premium Beauty News 2019. https://www.premiumbeautynews.com/en/germany-natural-and-organic,14771.

[18] Jørgensen C. (2020). Cosmetics Worldwide—Same Contents? A Comparative Study.

[19] Jensen L.M.M. Chemical of the Month: Iodopropynyl Butylcarbamate (IPBC). AllergyCertified 2017. https://allergycertified.com/blog/2017/11/22/iodopropynyl-butylcarbamate/.

[20] Hass U., Christiansen S., Petersen M.A., Boberg J., Andersson A.-M., Skakkebæk N.E., Bay K., Holbeck H., Lund Kinnberg K., Bjerregaard P. (2012). Evaluation of 22 SIN List 2.0 Substances According to the Danish Proposal on Criteria for Endocrine Disrupters.

[21] Becker L.C. (2017). Amended Safety Assessment of Glyoxal as Used in Cosmetics.

[22] Darbre P.D., Harvey P.W. (2014). Parabens can enable hallmarks and characteristics of cancer in human breast epithelial cells: A review of the literature with reference to new exposure data and regulatory status: Parabens and breast cancer. J. Appl. Toxicol..

[23] Berger K.P., Kogut K.R., Bradman A., She J., Gavin Q., Zahedi R., Parra K.L., Harley K.G. (2019). Personal care product use as a predictor of urinary concentrations of certain phthalates, parabens, and phenols in the HERMOSA study. J. Exp. Sci. Environ. Epidemiol..

[24] European Commission to Ban More CMRs in Cosmetics n.d. https://chemicalwatch.com/208245/european-commission-to-ban-more-cmrs-in-cosmetics.

[25] McCook J.P. (2016). Topical Products for the Aging Face. Clin. Plast. Surg..

[26] Boran R. (2018). Investigations of anti-aging potential of *Hypericum origanifolium* Willd. for skincare formulations. Ind. Crop. Prod..

[27] Pires J., Pinto P., Moreira N. (1994). Lameiros de Trás-Os-Montes. Perspectivas de Futuro Para Estas Pastagens de Montanha.

[28] Rosário M.C. (2004). O Sistema Agrário de Trás-Os-Montes e a modernidade sustentável. Gest. Desenvolv..

[29] Ribeiro J. (2000). Caracterização genérica da região vinhateira do Alto Douro. Douro—Estudos & Documentos.

[30] Cardoso J., Bessa M., Marado M. (1978). Carta dos Solos (III,I).

[31] Braga F.G., Carvalho L.M., Carvalho M.J., Guedes-Pinto H., Torres-Pereira J.M., Neto M.F., Monteiro A. (2002). Variation of the Anthocyanin Content in *Sambucus nigra* L. Populations Growing in Portugal. J. Herbs Spices Med. Plants.

[32] Trindade C., Valdiviesso T., de Brás Oliveira P. (2019). Caraterização Morfológica das Inflorescências de Variedades de Sambucus nigra L..

[33] Centro Nacional de Competências dos Frutos Secos (2020). Amêndoa. Estudi de Produção e Comercialização nas Terras de Trás-Os-Mntes.

[34] Cordeiro V., Monteiro A. (2002). Almond Growing in Trás-Os-Montes Region (Portugal). Acta Hortic..

[35] Portal do INE 2013. https://www.ine.pt/xportal/xmain?xpid=INE&xpgid=ine_indicadores&indOcorrCod=0000708&xlang=pt&contexto=bd&selTab=tab2.

[36] de Figueiredo T., Almeida A., Araújo J. (2002). Edaphic characteristics of olive-tree areas in the Trás-Os-Montes Region (Portugal): A map-based approach. Acta Hortic..

[37] Sousa M., Pererira C., Guerra J., Abade E. (2007). Caracterização de Castas Cultivadas na Região Vitivinícola de Trás-Os-Montes, Sub regiões de Chaves, Planalto Mirandês e Valpaços.

[38] Gonçalves S., Gaivão I. (2021). Assessment of antigenotoxic properties in natural ingredients common in the Trás-os-Montes region: A Phd project. ScienceOpen.

[39] Viapiana A., Wesolowski M. (2017). The Phenolic Contents and Antioxidant Activities of Infusions of *Sambucus nigra* L.. Plant Foods Hum. Nutr..

[40] Atkinson M.D., Atkinson E. (2002). *Sambucus nigra* L.: *Sambucus nigra*. J. Ecol..

[41] Charlebois D., Byers P.L., Finn C.E., Thomas A.L., Janick J. (2010). Elderberry: Botany, Horticulture, Potential. Horticultural Reviews.

[42] Mabey R. (1988). The Complete New Herbal. A Practical Guide to Herbal Living.

[43] Olejnik A., Kowalska K., Olkowicz M., Rychlik J., Juzwa W., Myszka K., Dembczyński R., Białas W. (2015). Anti-inflammatory effects of gastrointestinal digested *Sambucus nigra* L. fruit extract analysed in co-cultured intestinal epithelial cells and lipopolysaccharide-stimulated macrophages. J. Funct. Foods.

[44] Ciocoiu M., Badescu L., Badulescu O., Badescu M. (2012). Intervention of *Sambucus nigra* Polyphenolic Extract in Experimental Arterial Hypertension. Int. J. Med. Health Sci..

[45] Salvador Â., Król E., Lemos V., Santos S., Bento F., Costa C., Almeida A., Szczepankiewicz D., Kulczyński B., Krejpcio Z. (2016). Effect of Elderberry (*Sambucus nigra* L.) Extract Supplementation in STZ-Induced Diabetic Rats Fed with a High-Fat Diet. IJMS Int. J. Mol. Sci..

[46] Bhattacharya S., Christensen K.B., Olsen L.C.B., Christensen L.P., Grevsen K., Færgeman N.J., Kristiansen K., Young J.F., Oksbjerg N. (2013). Bioactive Components from Flowers of *Sambucus nigra* L. Increase Glucose Uptake in Primary Porcine Myotube Cultures and Reduce Fat Accumulation in *Caenorhabditis elegans*. J. Agric. Food Chem..

[47] Pilot Clinical Study on a Proprietary Elderberry Extract: Efficacy in Addressing Influenza Symptoms n.d. https://www.semanticscholar.org/paper/Pilot-Clinical-Study-on-a-Proprietary-Elderberry-%3A/367d1c92716b6be462f26dbfe6c223863dc78464.

[48] Jarzycka A., Lewińska A., Gancarz R., Wilk K.A. (2013). Assessment of extracts of Helichrysum arenarium, Crataegus monogyna, *Sambucus nigra* in photoprotective UVA and UVB; photostability in cosmetic emulsions. J. Photochem. Photobiol. B.

[49] Picon P.D., Picon R.V., Costa A.F., Sander G.B., Amaral K.M., Aboy A.L., Henriques A.T. (2010). Randomized clinical trial of a phytotherapic compound containing *Pimpinella anisum*, *Foeniculum vulgare*, *Sambucus nigra*, and *Cassia augustifolia* for chronic constipation. BMC Complement. Altern. Med..

[50] Neto M. (2007). Sabugueiro—Potencialidades. http://www.drapn.min-agricultura.pt/drapn/conteudos/cen_documentos/outros/Sabugueiro_Potencialidades.pdf.

[51] Młynarczyk K., Walkowiak-Tomczak D., Lysiak G. (2018). Bioactive properties of *Sambucus nigra* L. as a functional ingredient for food and pharmaceutical industry. J. Funct. Foods.

[52] Vulic J., Vracar L., Sumic Z. (2008). Chemical characteristics of cultivated elderberry fruit. Acta Period. Technol..

[53] Veberic R., Jakopic J., Stampar F., Schmitzer V. (2009). European elderberry (*Sambucus nigra* L.) rich in sugars, organic acids, anthocyanins and selected polyphenols. Food Chem..

[54] Dawidowicz A.L., Wianowska D., Baraniak B. (2006). The antioxidant properties of alcoholic extracts from *Sambucus nigra* L. (antioxidant properties of extracts). LWT Food Sci. Technol..

[55] Duymuş H.G., Göger F., Başer K.H.C. (2014). In vitro antioxidant properties and anthocyanin compositions of elderberry extracts. Food Chem..

[56] Espín J.C., Soler-Rivas C., Wichers H.J., García-Viguera C. (2000). Anthocyanin-Based Natural Colorants: A New Source of Antiradical Activity for Foodstuff. J. Agric. Food Chem..

[57] Obied H.K., Allen M.S., Bedgood D.R., Prenzler P.D., Robards K., Stockmann R. (2005). Bioactivity and Analysis of Biophenols Recovered from Olive Mill Waste. J. Agric. Food Chem..

[58] Imenšek N., Sem V., Kolar M., Ivančič A., Kristl J. (2021). The Distribution of Minerals in Crucial Plant Parts of Various Elderberry (*Sambucus* spp.) Interspecific Hybrids. Plants.

[59] Sidor A., Gramza-Michałowska A. (2015). Advanced research on the antioxidant and health benefit of elderberry (*Sambucus nigra*) in food—A review. J. Funct. Foods.

[60] Christensen L.P., Kaack K., Fretté X.C. (2008). Selection of elderberry (*Sambucus nigra* L.) genotypes best suited for the preparation of elderflower extracts rich in flavonoids and phenolic acids. Eur. Food Res. Technol..

[61] Mikulic-Petkovsek M., Samoticha J., Eler K., Stampar F., Veberic R. (2015). Traditional Elderflower Beverages: A Rich Source of Phenolic Compounds with High Antioxidant Activity. J. Agric. Food Chem..

[62] Galanakis C.M. (2011). Olive fruit dietary fiber: Components, recovery and applications. Trends Food Sci. Technol..

[63] Chen L., Hu J.Y., Wang S.Q. (2012). The role of antioxidants in photoprotection: A critical review. J. Am. Acad. Dermatol..

[64] Salvador Â.C., Silvestre A.J.D., Rocha S.M., Vijayakumar R., Raja S.S.S. (2018). Comprehensive Insight into the Elderflowers and Elderberries (*Sambucus nigra* L.) Mono and Sesquiterpenic Metabolites: Factors that Modulate Their Composition. Secondary Metabolites—Sources and Applications.

[65] Rodrigues F., Pimentel F.B., Oliveira M.B.P.P. (2015). Olive by-products: Challenge application in cosmetic industry. Ind. Crop. Prod..

[66] Bratu M., Doroftei E., Negreanu-Pirjol T., Hostina C., Porta S. (2012). Determination of Antioxidant Activity and Toxicity of *Sambucus nigra* Fruit Extract Using Alternative Methods. Food Technol. Biotechnol..

[67] Karpova I.S., Lylo V.V., Macewicz L.L., Kotsarenko K.V., Palchykovska L.G., Ruban T.O., Lukash L.L. (2015). Lectins of *Sambucus nigra* as Biologically Active and DNA-Protective Substances. Acta Hortic..

[68] Gaetano L., Calabrese G., Perrino E. (2012). The Origin and Distribution of the Olive Tree and Olive Crop.

[69] Newton C., Terral J.-F., Ivorra S. (2006). The Egyptian olive (*Olea europaea* subsp. *europaea* ) in the later first millennium BC: Origins and history using the morphometric analysis of olive stones. Antiquity.

[70] Ryan D., Robards K. (1998). Critical Review. Phenolic compounds in olives. Analyst.

[71] Grossi M., Lecce G.D., Toschi T.G., Ricco B. (2014). Fast and Accurate Determination of Olive Oil Acidity by Electrochemical Impedance Spectroscopy. IEEE Sens. J..

[72] Montaño A., Sánchez A.H., López-López A., de Castro A., Rejano L. (2010). Chemical Composition of Fermented Green Olives. Olives and Olive Oil in Health and Disease Prevention.

[73] Guo Z., Jia X., Zheng Z., Lu X., Zheng Y., Zheng B., Xiao J. (2018). Chemical composition and nutritional function of olive (*Olea europaea* L.): A review. Phytochem. Rev..

[74] Cirilli M., Bellincontro A., Urbani S., Servili M., Esposto S., Mencarelli F., Muleo R. (2016). On-field monitoring of fruit ripening evolution and quality parameters in olive mutants using a portable NIR-AOTF device. Food Chem..

[75] Elbir M., Es-Safi N.E., Amhoud A., Mbarki M. (2015). Characterization of phenolic compounds in olive stones of three moroccan varieties. Maderas Cienc. Tecnol..

[76] Fernandez-Bolanos J., Fernandez Diez M.J., Rivas Moreno M., Gil Serrano A., Perez Romero T. (1983). Sugars and polyois in green olives. III. Quantitative determination by gas-liquid chromatography. Grasas Aceites.

[77] Jilani H., Cilla A., Barberá R., Hamdi M. (2016). Improved bioaccessibility and antioxidant capacity of olive leaf (*Olea europaea* L.) polyphenols through biosorption on *Saccharomyces cerevisiae*. Ind. Crop. Prod..

[78] Talhaoui N., Taamalli A., Gómez-Caravaca A.M., Fernández-Gutiérrez A., Segura-Carretero A. (2015). Phenolic compounds in olive leaves: Analytical determination, biotic and abiotic influence, and health benefits. Food Res. Int..

[79] Gouvinhas I., Machado N., Sobreira C., Domínguez-Perles R., Gomes S., Rosa E., Barros A. (2017). Critical Review on the Significance of Olive Phytochemicals in Plant Physiology and Human Health. Molecules.

[80] Ahmed A.M., Rabii N.S., Garbaj A.M., Abolghait S.K. (2014). Antibacterial effect of olive (*Olea europaea* L.) leaves extract in raw peeled undeveined shrimp (*Penaeus semisulcatus*). Int. J. Vet. Sci. Med..

[81] Al Okbi S., Hassan Z., El Mazar M., Ammar N., Abou Elkassem L., El Bakry H. (2011). Diuretic activity of Olive (*Olea europaea* L.). Planta Med..

[82] Khan M., Panchal S., Vyas N., Butani A., Kumar V. (2006). *Olea europaea*: A phyto-pharmacological review. Pharmacogn. Rev..

[83] Khayyal M., El-Ghazaly M., Abdallah D., Nassar N., Okpanyi S., Kreuter M.-H. (2011). Blood Pressure Lowering Effect of an Olive Leaf Extract {*Olea europaed*) in L-NAME Induced Hypertension in Rats. Arzneimittelforschung.

[84] Mousa H.M., Farahna M., Ismail M.S., Al-Hassan A.A., Ammar A.S., Abdel-Salam A.M. (2014). Anti-Diabetic Effect of Olive Leaves Extract in Alloxan-Diabetic Rats. J. Agric. Vet. Sci..

[85] Erbay Z., Icier F. (2010). The Importance and Potential Uses of Olive Leaves. Food Rev. Int..

[86] Autoridade de Segurança Alimentar e Económica Azeites e sua Classificação. https://www.asae.gov.pt/newsletter2/asaenews-n-108-julho-2017/azeites-e-sua-classificacao-.aspx.

[87] FoodData Central, n.d. https://fdc.nal.usda.gov/fdc-app.html#/food-details/748608/nutrients.

[88] Huang Z.-R., Lin Y.-K., Fang J.-Y. (2009). Biological and Pharmacological Activities of Squalene and Related Compounds: Potential Uses in Cosmetic Dermatology. Molecules.

[89] Scoditti E., Capurso C., Capurso A., Massaro M. (2014). Vascular effects of the Mediterranean diet—Part II: Role of omega-3 fatty acids and olive oil polyphenols. Vascul. Pharmacol..

[90] Trapani G., Vagliano L., Giribaldi M., Cavallarin L., Coscia A. (2017). Olive oil: Maternal and pediatric health. J. Pediatr. Neonatal Individ. Med..

[91] Coletta J.M., Bell S.J., Roman A.S. (2010). Omega-3 Fatty Acids and Pregnancy. Rev. Obstet. Gynecol..

[92] Roncero-Martín R., Aliaga Vera I., Moreno-Corral L., Moran J., Lavado-Garcia J., Pedrera-Zamorano J., Pedrera-Canal M. (2018). Olive Oil Consumption and Bone Microarchitecture in Spanish Women. Nutrients.

[93] Widmer R.J., Freund M.A., Flammer A.J., Sexton J., Lennon R., Romani A., Mulinacci N., Vinceri F.F., Lerman L.O., Lerman A. (2013). Beneficial effects of polyphenol-rich olive oil in patients with early atherosclerosis. Eur. J. Nutr..

[94] Assy N., Nassar F., Nasser G., Grosovski M. (2009). Olive oil consumption and non-alcoholic fatty liver disease. WJG World J. Gastroenterol..

[95] Yubero-Serrano E.M., Lopez-Moreno J., Gomez-Delgado F., Lopez-Miranda J. (2019). Extra virgin olive oil: More than a healthy fat. Eur. J. Clin. Nutr..

[96] Brzosko S., Curtis A.D., Murzilli S., Gaetano G.D., Donati M.B., Iacoviello L. (2002). Effect of extra virgin olive oil on experimental thrombosis and primary hemostasis in rats. Nutr. Metab. Cardiovasc. Dis. NMCD.

[97] Lauretti E., Nenov M., Dincer O., Iuliano L., Praticò D. (2020). Extra virgin olive oil improves synaptic activity, short-term plasticity, memory, and neuropathology in a tauopathy model. Aging Cell.

[98] Condelli N., Caruso M.C., Galgano F., Russo D., Milella L., Favati F. (2015). Prediction of the antioxidant activity of extra virgin olive oils produced in the Mediterranean area. Food Chem..

[99] Genders R. (1987). Natural Beauty—The Practical Guide to Wild Flower Cosmetics.

[100] Smaoui S., Hlima H.B., Jarraya R., Kamoun N.G., Ellouze R., Damak M. (2012). Cosmetic emulsion from virgin olive oil: Formulation and bio-physical evaluation. Afr. J. Biotechnol..

[101] Angerhofer C.K., Maes D., Giacomoni P.U. (2009). The Use of Natural Compounds and Botanicals in the Development of Anti-Aging Skin Care Products. Skin Aging Handbook.

[102] Viola P., Viola M. (2009). Virgin olive oil as a fundamental nutritional component and skin protector. Clin. Dermatol..

[103] Lin M.-H., Khnykin D. (2014). Fatty acid transporters in skin development, function and disease. Biochim. Biophys. Acta BBA—Mol. Cell Biol. Lipids.

[104] Aly F.M., Kotb A.M., Haridy M.A.M., Hammad S. (2018). Impacts of fullerene C60 and virgin olive oil on cadmium-induced genotoxicity in rats. Sci. Total Environ..

[105] Khalil S., Awad A., Elewa Y. (2013). Antidotal impact of extra virgin olive oil against genotoxicity, cytotoxicity and immunotoxicity induced by hexavalent chromium in rat. Int. J. Vet. Sci. Med..

[106] Ladizinsky G. (1999). On the Origin of Almond. Genet. Resour. Crop Evol..

[107] Albala K. (2009). Almonds Along the Silk Road: The Exchange and Adaptation of Ideas from West to East. Petits Propos Culin..

[108] Offeman R.D., Holtman K.M., Covello K.M., Orts W.J. (2014). Almond hulls as a biofuels feedstock: Variations in carbohydrates by variety and location in California. Ind. Crop. Prod..

[109] Richardson D.P., Astrup A., Cocaul A., Ellis P. (2009). The nutritional and health benefits of almonds: A healthy food choice. Food Sci. Technol. Bull. Funct. Foods.

[110] Beltrán Sanahuja A., Maestre Pérez S.E., Grané Teruel N., Valdés García A., Prats Moya M.S. (2021). Variability of Chemical Profile in Almonds (*Prunus dulcis*) of Different Cultivars and Origins. Foods.

[111] Socias R., Kodad O., Alonso J.M., Gradziel T.M., Janick J. (2008). Almond Quality: A Breeding Perspective. Horticultural Reviews.

[112] Roncero J.M., Álvarez-Ortí M., Pardo-Giménez A., Rabadán A., Pardo J.E. (2020). Review about Non-Lipid Components and Minor Fat-Soluble Bioactive Compounds of Almond Kernel. Foods.

[113] Kodad O., Alonso J.M., Espiau M.T., Estopañán G., Juan T., Rafel Socias i Company (2011). Chemometric Characterization of Almond Germplasm: Compositional Aspects Involved in Quality and Breeding. J. Am. Soc. Hortic. Sci..

[114] Fernandes G.D., Gómez-Coca R.B., Pérez-Camino M.d.C., Moreda W., Barrera-Arellano D. (2017). Chemical Characterization of Major and Minor Compounds of Nut Oils: Almond, Hazelnut, and Pecan Nut. J. Chem..

[115] Fernández-Cuesta Á., Kodad O., Velasco L., Rafel Socias i Company (2012). Phytosterol Variability in Almond Germplasm. J. Am. Soc. Hortic. Sci..

[116] Garrido I., Monagas M., Gómez-Cordovés C., Bartolomé B. (2008). Polyphenols and Antioxidant Properties of Almond Skins: Influence of Industrial Processing. J. Food Sci..

[117] Xiao L., Lee J., Zhang G., Ebeler S.E., Wickramasinghe N., Seiber J., Mitchell A.E. (2014). HS-SPME GC/MS characterization of volatiles in raw and dry-roasted almonds (*Prunus dulcis*). Food Chem..

[118] Franklin L.M., Mitchell A.E. (2019). Review of the Sensory and Chemical Characteristics of Almond (*Prunus dulcis*) Flavor. J. Agric. Food Chem..

[119] (1983). 4 Final Report on the Safety Assessment of Sweet Almond Oil and Almond Meal. J. Am. Coll. Toxicol..

[120] Deuschle V.C.K.N., Deuschle R.A.N., Bortoluzzi M.R., Athayde M.L. (2015). Physical chemistry evaluation of stability, spreadability, in vitro antioxidant, and photo-protective capacities of topical formulations containing *Calendula officinalis* L. leaf extract. Braz. J. Pharm. Sci..

[121] Ngoc L.T.N., Tran V.V., Moon J.Y., Chae M., Park D., Lee Y.C. (2019). Recent Trends of Sunscreen Cosmetic: An Update Review. Cosmetics.

[122] Jia X., Li N., Zhang W., Zhang X., Lapsley K., Huang G., Blumberg J., Ma G., Chen J. (2006). A Pilot Study on the Effects of Almond Consumption on DNA Damage and Oxidative Stress in Smokers. Nutr. Cancer.

[123] Zhang X., Xiang Q., Cui W., Jia X., Li N. (2011). Evaluation of the in vitro and in vivo Genotoxicity of Almond Skins. Biomed. Environ. Sci..

[124] Lux S., Scharlau D., Schlörmann W., Birringer M., Glei M. (2012). In vitro fermented nuts exhibit chemopreventive effects in HT29 colon cancer cells. Br. J. Nutr..

[125] Briviba K., Gräf V., Walz E., Guamis B., Butz P. (2016). Ultra high pressure homogenization of almond milk: Physico-chemical and physiological effects. Food Chem..

[126] Schlörmann W., Fischer S., Saupe C., Dinc T., Lorkowski S., Glei M. (2018). Influence of roasting on the chemopreventive potential of in vitro fermented almonds in LT97 colon adenoma cells. Int. J. Food Sci. Nutr..

[127] Martin M.E., Grao-Cruces E., Millan-Linares M.C., Montserrat-de la Paz S. (2020). Grape (*Vitis vinifera* L.) Seed Oil: A Functional Food from the Winemaking Industry. Foods.

[128] Myles S., Boyko A.R., Owens C.L., Brown P.J., Grassi F., Aradhya M.K., Prins B., Reynolds A., Chia J.-M., Ware D. (2011). Genetic structure and domestication history of the grape. Proc. Natl. Acad. Sci. USA.

[129] Vindimas em Portugal: Uma Tradição que Perdura. Clube de Vinhos 2010. https://clubedevinhos.com/artigos/vindimas-portugal-tradicao-que-perdura.

[130] De Rosso M., Panighel A., Carraro R., Padoan E., Favaro A., Dalla Vedova A., Flamini R. (2010). Chemical Characterization and Enological Potential of Raboso Varieties by Study of Secondary Grape Metabolites. J. Agric. Food Chem..

[131] Mota A., Pinto J., Fartouce I., Correia M.J., Costa R., Carvalho R., Aires A., Oliveira A.A. (2018). Chemical profile and antioxidant potential of four table grape (*Vitis vinifera*) cultivars grown in Douro region, Portugal. Ciênc. e Téc. Vitiviníc..

[132] Shinagawa F.B., de Santana F.C., Torres L.R.O., Mancini-Filho J. (2015). Grape seed oil: A potential functional food?. Food Sci. Technol..

[133] Zielinski E. (2020). The Healing Power of Essential Oils.

[134] Ramos S. (2019). Os Segredos da Aromaterapia: Um Guia Completo de Óleos Essenciais Para a sua Saúde, Beleza e Bem-Estar.

[135] Cavaliere C., Foglia P., Gubbiotti R., Sacchetti P., Samperi R., Laganà A. (2008). Rapid-resolution liquid chromatography/mass spectrometry for determination and quantitation of polyphenols in grape berries. Rapid Commun. Mass Spectrom..

[136] Garavaglia J., Markoski M.M., Oliveira A., Marcadenti A. (2016). Grape Seed Oil Compounds: Biological and Chemical Actions for Health. Nutr. Metab. Insights.

[137] Ullah A. (2018). Physiochemical properties and fatty acid profile of grapes available in Peshawar city. PAB Pure Appl. Biol..

[138] Assumpção C.F., Nunes I.L., Mendonça T.A., Bortolin R.C., Jablonski A., Flôres S.H., de Oliveira Rios A. (2016). Bioactive Compounds and Stability of Organic and Conventional Vitis labrusca Grape Seed Oils. J. Am. Oil Chem. Soc..

[139] Fernandes L., Casal S., Cruz R., Pereira J.A., Ramalhosa E. (2013). Seed oils of ten traditional Portuguese grape varieties with interesting chemical and antioxidant properties. Food Res. Int..

[140] Shinagawa F.B., de Santana F.C., Mancini-Filho J. (2015). Efeito do óleo de semente de uva prensado a frio nos marcadores bioquímicos e perfil inflamatório de ratos. Rev. Nutr..

[141] Huang S., Yang N., Liu Y., Gao J., Huang T., Hu L., Zhao J., Li Y., Li C., Zhang X. (2012). Grape seed proanthocyanidins inhibit colon cancer-induced angiogenesis through suppressing the expression of VEGF and Angl. Int. J. Mol. Med..

[142] Ky I., Teissedre P.-L. (2015). Characterisation of Mediterranean Grape Pomace Seed and Skin Extracts: Polyphenolic Content and Antioxidant Activity. Molecules.

[143] Monrad J.K., Howard L.R., King J.W., Srinivas K., Mauromoustakos A. (2010). Subcritical Solvent Extraction of Anthocyanins from Dried Red Grape Pomace. J. Agric. Food Chem..

[144] Dwyer K., Hosseinian F., Rod M. (2014). The Market Potential of Grape Waste Alternatives. JFR J. Food Res..

[145] Hübner A.A., Sarruf F.D., Oliveira C.A., Neto A.V., Fischer D.C.H., Kato E.T.M., Lourenço F.R., Baby A.R., Bacchi E.M. (2020). Safety and Photoprotective Efficacy of a Sunscreen System Based on Grape Pomace (*Vitis vinifera* L.) Phenolics from Winemaking. Pharmaceutics.

[146] Fiume M.M., Bergfeld W.F., Belsito D.V., Hill R.A., Klaassen C.D., Liebler D.C., Marks J.G., Shank R.C., Slaga T.J., Snyder P.W. (2014). Safety Assessment of *Vitis vinifera* (Grape)-Derived Ingredients as Used in Cosmetics. Int. J. Toxicol..

[147] de Campos L.M.A.S., Leimann F.V., Pedrosa R.C., Ferreira S.R.S. (2008). Free radical scavenging of grape pomace extracts from Cabernet sauvingnon (*Vitis vinifera*). Bioresour. Technol..

[148] Ratz-Łyko A., Arct J. (2019). Resveratrol as an active ingredient for cosmetic and dermatological applications: A review. J. Cosmet. Laser Ther..

[149] Saewan N., Jimtaisong A. (2015). Natural products as photoprotection. J. Cosmet. Dermatol..

[150] Saraf S., Kaur C. (2010). Phytoconstituents as photoprotective novel cosmetic formulations. Pharmacogn. Rev..

[151] Lorencini M., Brohem C.A., Dieamant G.C., Zanchin N.I.T., Maibach H.I. (2014). Active ingredients against human epidermal aging. Ageing Res. Rev..

[152] Radice M., Manfredini S., Ziosi P., Dissette V., Buso P., Fallacara A., Vertuani S. (2016). Herbal extracts, lichens and biomolecules as natural photo-protection alternatives to synthetic UV filters. A systematic review. Fitoterapia.

[153] Edenharder R., Sager J.W., Glatt H., Muckel E., Platt K.L. (2002). Protection by beverages, fruits, vegetables, herbs, and flavonoids against genotoxicity of 2-acetylaminofluorene and 2-amino-1-methyl-6-phenylimidazo[4,5-b]pyridine (PhIP) in metabolically competent V79 cells. Mutat. Res. Genet. Toxicol. Environ. Mutagen..

[154] Llópiz N., Puiggròs F., Céspedes E., Arola L., Ardévol A., Bladé C., Salvadó M.J. (2004). Antigenotoxic Effect of Grape Seed Procyanidin Extract in Fao Cells Submitted to Oxidative Stress. J. Agric. Food Chem..

[155] de Rezende A.A.A., Graf U., da Rosa Guterres Z., Kerr W.E., Spanó M.A. (2009). Protective effects of proanthocyanidins of grape (*Vitis vinifera* L.) seeds on DNA damage induced by Doxorubicin in somatic cells of *Drosophila melanogaster*. Food Chem. Toxicol..

[156] Anter J., de Abreu-Abreu N., Fernández-Bedmar Z., Villatoro-Pulido M., Alonso-Moraga Á., Muñoz-Serrano A. (2011). Targets of Red Grapes: Oxidative Damage of DNA and Leukaemia Cells. Nat. Prod. Commun..

[157] de Moura C.F.G., Ribeiro F.A.P., de Jesus G.P.P., da Silva V.H.P., Oshima C.T.F., Gollücke A.P.B., Aguiar O., Ribeiro D.A. (2014). Antimutagenic and antigenotoxic potential of grape juice concentrate in blood and liver of rats exposed to cadmium. Environ. Sci. Pollut. Res..

[158] Corredor Z., Rodríguez-Ribera L., Coll E., Montañés R., Diaz J.M., Ballarin J., Marcos R., Pastor S. (2016). Unfermented grape juice reduce genomic damage on patients undergoing hemodialysis. Food Chem. Toxicol..

[159] D’Abrosca B., Lavorgna M., Scognamiglio M., Russo C., Graziani V., Piscitelli C., Fiorentino A., Isidori M. (2017). 2D-NMR investigation and in vitro evaluation of antioxidant, antigenotoxic and estrogenic/antiestrogenic activities of strawberry grape. Food Chem. Toxicol..

[160] Sierra L.M., Gaivão I. (2014). The Control of Hydrophobic Compound Exposure in In Vitro Tests for Genotoxicity. Genotoxicity and DNA Repair.

[161] Liao W., McNutt M.A., Zhu W.-G. (2009). The comet assay: A sensitive method for detecting DNA damage in individual cells. Methods.

[162] Kumaravel T.S., Vilhar B., Faux S.P., Jha A.N. (2009). Comet Assay measurements: A perspective. Cell Biol. Toxicol..

[163] Singh N.P., McCoy M.T., Tice R.R., Schneider E.L. (1988). A simple technique for quantitation of low levels of DNA damage in individual cells. Exp. Cell Res..

[164] Hartwig A. (1996). Sensitive analysis of oxidative DNA damage in mammalian cells: Use of the bacterial Fpg protein in combination with alkaline unwinding. Toxicol. Lett..

[165] Fahim M., Ahmed A., Hussain S. (2017). Single cell gel electrophoresis and its applications in different fields. MOJ Bioequiv Availab.

[166] Gaivão I., Comendador M.A. (1996). The w/w+ somatic mutation and recombination test (SMART) of Drosophila melanogaster for detecting reactive oxygen species: Characterization of 6 strains. Mutat. Res. Environ. Mutagen. Relat. Subj..

[167] Ferreira J., Marques A., Abreu H., Pereira R., Rego A., Pacheco M., Gaivão I. (2019). Red seaweeds *Porphyra umbilicalis* and *Grateloupia turuturu* display antigenotoxic and longevity-promoting potential in *Drosophila melanogaster*. Eur. J. Phycol..

[168] Graf U., van Schaik N. (1992). Improved high bioactivation cross for the wing somatic mutation and recombination test in *Drosophila melanogaster*. Mutat. Res. Environ. Mutagen. Relat. Subj..

[169] Han S.-J. (2015). High-throughput, in vivo genotoxicity testing: An automated readout system for the Somatic Mutation and Recombination Test (SMART). PLoS ONE.

[170] Fenech M., Morley A.A. (1985). Measurement of micronuclei in lymphocytes. Mutat. Res. Environ. Mutagen. Relat. Subj..

[171] Sommer S., Buraczewska I., Kruszewski M. (2020). Micronucleus Assay: The State of Art, and Future Directions. IJMS Int. J. Mol. Sci..

[172] Terradas M., Martín M., Tusell L., Genescà A. (2010). Genetic activities in micronuclei: Is the DNA entrapped in micronuclei lost for the cell?. Mutat. Res. Rev. Mutat. Res..

[173] D’Costa A., Praveen Kumar M.K., Shyama S.K. (2019). Genotoxicity assays. Advances in Biological Science Research.

[174] Emam A.N., Girgis E., Khalil W.K.B., Mohamed M.B. (2014). Toxicity of Plasmonic Nanomaterials and Their Hybrid Nanocomposites. Advances in Molecular Toxicology.

[175] Fenech M. (1997). The advantages and disadvantages of the cytokinesis-block micronucleus method. Mutat. Res. Genet. Toxicol. Environ. Mutagen..

[176] Scientific Committee on Emerging and Newly Identified Health Risks (2009). Health Effects of Exposure to EMF.

[177] Registre M., Proudlock R. (2016). The In Vitro Chromosome Aberration Test. Genetic Toxicology Testing.

[178] Chromosome Aberration Test In Vitro. Eurofins Scientific n.d. https://www.eurofins.de/biopharma-product-testing-en/validated-standard-testing-methods/chromosome-aberration-test/.

[179] Kirkland D.J. (1992). Chromosomal aberration tests in vitro: Problems with protocol design and interpretation of results. Mutagenesis.

[180] Gatehouse D., Haworth S., Cebula T., Gocke E., Kier L., Matsushima T., Melcion C., Nohmi T., Ohta T., Venitt S. (1994). Recommendations for the performance of bacterial mutation assays. Mutat. Res. Environ. Mutagen. Relat. Subj..

[181] Ames B.N., McCann J., Yamasaki E. (1975). Methods for detecting carcinogens and mutagens with the salmonella/mammalian-microsome mutagenicity test. Mutat. Res. Environ. Mutagen. Relat. Subj..

[182] Center for Food Safety and Applied Nutrition, Office of Food Additive Safety (2019). Redbook 2000: IV.C.1.a. Bacterial Reverse Mutation Test.

[183] Pillco A., de la Peña E., Sierra L.M., Gaivão I. (2014). Ames Test (Bacterial Reverse Mutation Test): Why, When, and How to Use. Genotoxicity and DNA Repair.

[184] Mortelmans K., Zeiger E. (2000). The Ames Salmonella/microsome mutagenicity assay. Mutat. Res. Fundam. Mol. Mech. Mutagen..

[185] Rand M.D. (2010). Drosophotoxicology: The growing potential for Drosophila in neurotoxicology. Neurotoxicol. Teratol..

[186] Rand M.D., Lowe J.A., Mahapatra C.T. (2012). Drosophila CYP6g1 and its human homolog CYP3A4 confer tolerance to methylmercury during development. Toxicology.

[187] Sudati J.H., Vieira F.A., Pavin S.S., Dias G.R.M., Seeger R.L., Golombieski R., Athayde M.L., Soares F.A., Rocha J.B.T., Barbosa N.V. (2013). *Valeriana officinalis* attenuates the rotenone-induced toxicity in *Drosophila melanogaster*. NeuroToxicology.

[188] Abolaji A., Kamdem J.P., Farombi O., Rocha J.B. (2013). Drosophila melanogaster as a Promising Model Organism in Toxicological Studies: A Mini Review. Arch. Basic Appl. Med..

[189] Piper M.D.W., Skorupa D., Partridge L. (2005). Diet, metabolism and lifespan in Drosophila. Exp. Gerontol..

[190] Lushchak O.V., Rovenko B.M., Gospodaryov D.V., Lushchak V.I. (2011). Drosophila melanogaster larvae fed by glucose and fructose demonstrate difference in oxidative stress markers and antioxidant enzymes of adult flies. Comp. Biochem. Physiol. Part A Mol. Integr. Physiol..

[191] Sheshadri D., Shetty N., Shivanandappa T., Ramesh S.R. (2014). Oxidative stress and longevity: Evidence from a long-lived strain of Drosophila melanogaster. Drosoph. Inf. Serv..

[192] Møller P., Möller L., Godschalk R.W.L., Jones G.D.D. (2010). Assessment and reduction of comet assay variation in relation to DNA damage: Studies from the European Comet Assay Validation Group. Mutagenesis.

[193] López-Romero D., Izquierdo-Vega J., Morales-González J., Madrigal-Bujaidar E., Chamorro-Cevallos G., Sánchez-Gutiérrez M., Betanzos-Cabrera G., Alvarez-Gonzalez I., Morales-González Á., Madrigal-Santillán E. (2018). Evidence of Some Natural Products with Antigenotoxic Effects. Part 2: Plants, Vegetables, and Natural Resin. Nutrients.

[194] Melo K.M., Fascineli M.L., Milhomem-Paixão S.S.R., Grisolia C.K., Santos A.S., Salgado H.L.C., Muehlmann L.A., Azevedo R.B., Pieczarka J.C., Nagamachi C.Y. (2018). Evaluation of the Genotoxic and Antigenotoxic Effects of Andiroba (*Carapa guianensis* Aublet) Oil and Nanoemulsion on Swiss Mice. J. Nanomater..

[195] Cruz M., Antunes P., Paulo L., Ferreira A.M., Cunha A., Almeida-Aguiar C., Oliveira R. (2016). Antioxidant and dual dose-dependent antigenotoxic and genotoxic properties of an ethanol extract of propolis. RSC Adv..

